# The Mechanisms Responsible for N Deficiency in Well-Watered Wheat Under Elevated CO_2_

**DOI:** 10.3389/fpls.2022.801443

**Published:** 2022-02-16

**Authors:** Jinjie Fan, Moshe Halpern, Yangliu Yu, Qiang Zuo, Jianchu Shi, Yuchuan Fan, Xun Wu, Uri Yermiyahu, Jiandong Sheng, Pingan Jiang, Alon Ben-Gal

**Affiliations:** ^1^Key Laboratory of Plant-Soil Interactions, Ministry of Education, Key Laboratory of Arable Land Conservation (North China), Ministry of Agriculture, College of Land Science and Technology, China Agricultural University, Beijing, China; ^2^Soil, Water and Environmental Sciences, Agricultural Research Organization, Gilat Research Center, Mobile Post Negev, Israel; ^3^College of Resources and Environment, Xinjiang Agricultural University, Ürümqi, China

**Keywords:** elevated CO_2_ concentration, nitrogen deficiency, nitrogen dilution, nitrogen uptake, photosynthesis, transpiration

## Abstract

Elevated CO_2_ concentration [e(CO_2_)] often promotes plant growth with a decrease in tissue N concentration. In this study, three experiments, two under hydroponic and one in well-watered soil, including various levels or patterns of CO_2_, humidity, and N supply were conducted on wheat (*Triticum aestivum* L.) to explore the mechanisms of e[CO_2_]-induced N deficiency (ECIND). Under hydroponic conditions, N uptake remained constant even as transpiration was limited 40% by raising air relative humidity and only was reduced about 20% by supplying N during nighttime rather than daytime with a reduction of 85% in transpiration. Compared to ambient CO_2_ concentration, whether under hydroponic or well-watered soil conditions, and whether transpiration was kept stable or decreased to 12%, e[CO_2_] consistently led to more N uptake and higher biomass, while lower N concentration was observed in aboveground organs, especially leaves, as long as N supply was insufficient. These results show that, due to compensation caused by active uptake, N uptake can be uncoupled from water uptake under well-watered conditions, and changes in transpiration therefore do not account for ECIND. Similar or lower tissue ${\text{NO}}_{3}^{-}$-N concentration under e[CO_2_] indicated that ${\text{NO}}_{3}^{-}$ assimilation was not limited and could therefore also be eliminated as a major cause of ECIND under our conditions. Active uptake has the potential to bridge the gap between N taken up passively and plant demand, but is limited by the energy required to drive it. Compared to ambient CO_2_ concentration, the increase in N uptake under e[CO_2_] failed to match the increase of carbohydrates, leading to N dilution in plant tissues, the apparent dominant mechanism explaining ECIND. Lower N concentration in leaves rather than roots under e[CO_2_] validated that ECIND was at least partially also related to changes in resource allocation, apparently to maintain root uptake activity and prevent more serious N deficiency.

## Introduction

Global atmospheric CO_2_ concentration has increased from 280 μmol mol^–1^ before the industrial revolution to 410 μmol mol^––1^ in 2020^1^, and is expected to double by the end of this century due to human activity (IPCC, 2007). Elevated CO_2_ concentration [e(CO_2_)] generally decreases leaf stomatal conductance and transpiration rate and increases the photosynthetic rate and biomass accumulation for C3 plants. On the other hand, e[CO_2_] often leads to a decrease in tissue N concentration, especially under conditions with low N availability (Ainsworth and Rogers, 2007; Bloom et al., 2010). An important consequence of N deficiency is decrease of proteins in the edible parts of plants, which can have profound effects on food quality (Loladze, 2002; Ziska et al., 2004; Li Y. et al., 2018). For wheat and other C3 plants growing under e[CO_2_], N concentrations in leaves and grain generally decrease 10–15% compared to those under ambient CO_2_ concentration (Conroy and Hocking, 1993; Stitt and Krapp, 1999; Wang et al., 2021). Additionally, photosynthetic acclimation to long term exposure to e[CO_2_], which weakens the positive effects of e[CO_2_] on plant photosynthesis and yield, is partially the result of decrease in leaf N concentration (Ainsworth and Long, 2005; Bloom et al., 2010; Dong et al., 2017, 2018).

Although e[CO_2_]-induced N deficiency (ECIND) has received quite a bit of attention, no primary causative factor for this phenomenon has been agreed upon (Bloom et al., 2014; Halpern et al., 2019). Many hypotheses have been proposed to account for ECIND. Based on the positive correlation between plant transpiration and N uptake (Dalton et al., 1975; Schoups and Hopmans, 2002; Shi et al., 2013), one possibility is that e[CO_2_] reduces plant transpiration, which in turn reduces N uptake (Kanemoto et al., 2009; McGrath and Lobell, 2013; Houshmandfar et al., 2018). A second hypothesis states that e[CO_2_] reduces ${\text{NO}}_{3}^{-}$ assimilation and thus organic N compounds through a variety of physiological mechanisms, including decreased availability of reductants, due to limited photorespiration and increased competition for reductants in the chloroplast stroma between carbon fixation and ${\text{NO}}_{3}^{-}$ reduction (Bloom et al., 2002, 2010, 2014). A third hypothesis is that ECIND is simply caused by the dilution of N in plant tissues, which results from the inability of roots to take up enough N from the growth medium to keep up with the growth accelerated by e[CO_2_] (Conroy and Hocking, 1993; Stitt and Krapp, 1999). Lastly, it is possible that changes in resource allocation under e[CO_2_] can also lead to N deficiency in aboveground organs (Kanemoto et al., 2009; McGrath and Lobell, 2013; Igarashi et al., 2021). The existence of clear evidence for each of these mechanisms, combined with the fact that none of them is theoretically mutually exclusive, indicates that each may explain ECIND, and that the dominant mechanism may depend on the circumstances.

To be taken up by a plant, N is first transported from the surrounding medium to the root surfaces and subsequently is moved into the roots. From medium to root surfaces, N transport is dominated by physical mechanisms such as convection based on transpiration-driven water flow and hydrodynamic dispersion, including molecular diffusion and mechanical dispersion and the distending of solutes transported with the flow of an advecting fluid. Transport of N from root surfaces into roots, also driven by physical mechanisms, is additionally governed by plant physiological mechanisms, with a distinction made between passive and active uptake from a macroscopic perspective (Jungk, 2002; Darrah et al., 2006). Passive uptake occurs as N is transported along the energy gradient from root surfaces into the roots, a physical process. When passive uptake cannot satisfy plant N demand, active uptake induced by physiological mechanisms will play a role to narrow this gap by transporting N into roots against the energy gradient (Epstein and Bloom, 2005; Shi et al., 2013; Li S. et al., 2018).

It is unclear whether transpiration is coupled with N uptake under well-watered conditions, and whether the decrease in transpiration under e[CO_2_] contributes to ECIND. On one hand, in a well-mixed hydroponic solution, there should be no impediment for the ${\text{NO}}_{3}^{-}$ ions to reach the root surface. In fact, Schulze and Bloom (1984) showed that transpiration and N uptake was uncoupled in radishes and tomatoes grown in a hydroponic system. According to this reasoning, ECIND that occurs under hydroponic conditions must be the result of other mechanisms, such as reduced ${\text{NO}}_{3}^{-}$ assimilation, N dilution, or changes in resource allocation. On the other hand, a reduction in transpiration could decrease the rate of passive transport into the roots and increase the energetic cost of N uptake (Epstein and Bloom, 2005; Shi et al., 2013). Thus the reduction in transpiration in e[CO_2_] should contribute to ECIND. This question was investigated using a series of hydroponic experiments. We further investigated how N uptake is affected by transpiration rate in well-watered soil conditions, in which hydrodynamic dispersion should be dominant over mass flow in the path from the soil to the root surface, making it similar, but not identical to, hydroponic conditions.

## Materials and Methods

In an initial experiment (Exp. 1), wheat was cultured hydroponically in growth chambers with various supply levels of atmospheric CO_2_ and solution N to explore the relationships between transpiration, N uptake, resource allocation, and ECIND. A second experiment (Exp. 2) was conducted to compare or validate the results obtained under hydroponic conditions by culturing wheat under well-watered soil conditions with varied CO_2_ and N supply levels. In a third trial (Exp. 3), wheat transpiration and N uptake in a hydroponic system were manipulated by altering relative humidity in growth chambers along with level and timing of N supply to investigate their interrelations without any variation of CO_2_.

### Exp. 1: Hydroponic—N × CO_2_

An experiment of hydroponically cultured spring wheat (*Triticum aestivum* L. cv. Yunmai 42) was conducted in three artificial climate chambers with constant conditions set as: photosynthetic photon flux density of 400 μmol m^–2^ s^–1^ over plants from 08:00 to 20:00, day/night temperature of 30/20°C ± 1°C; and relative humidity of 40% ± 5%. The surface was sterilized with 0.83 mmol cm^–3^ hydrogen peroxide solution for 0.5 h and then washed three times with deionized water. The seeds were soaked for 4 h in saturated CaSO_4_ solution and germinated on moist filter paper in darkness for 2 days at 25°C. On May 22, 2014, the seedlings were planted into quartz sand and irrigated with sufficient half-strength Hoagland solution (Shangguan et al., 2000), in which the concentrations of major elements were (mg cm^–3^): ${\text{NO}}_{3}^{-}$-N, 0.105; ${\text{SO}}_{4}^{2-}$-S, 0.032; ${\text{H}}_{2}{\text{PO}}_{4}^{-}$-P, 0.016; Mg^2+^, 0.024; K^+^, 0.117; Ca^2+^, 0.100. The concentrations of microelements in the solution were (10^–6^mg cm^–3^): Cu, 63.6; Zn, 156.9; Mn, 109.8; B, 10.8; Fe, 1675.5; Mo, 20.2 (Watkin et al., 1998).

From each of the three chambers with an initial CO_2_ concentration of 400 ± 50 μmol mol^–1^, 15 containers (25.5 cm in diameter and 10 cm in height) were prepared. Each of them was filled with 13 L modified half-strength Hoagland solution and covered with a board, in which 10 holes (2 cm in diameter) were bored symmetrically. With ${\text{NO}}_{3}^{-}$-N concentrations of 0.014 (N1), 0.028 (N2), 0.056 (N3), 0.105 (N4), and 0.140 (N5) mg cm^–3^ in the nutrient solution, 5 N treatments were set with 3 duplications, in which the pH value of 7.0 was adjusted by NaOH solution. On June 6, 2014 (14 days after planting, DAP), wheat seedlings were transplanted into the containers, and each seedling was fixed in a hole with sponge. Nutrient solution was replaced every 4 days and fresh air was injected continuously with a compressor.

At 08:00 on June 21, 2014 (29 DAP), the CO_2_ concentration in one of the three chambers, chosen randomly, was elevated and then maintained at 625 ± 50 μmol mol^–1^ and in a second chamber raised and maintained at 850 ± 50 μmol mol^–1^. Sampling was initiated on 29 DAP and then conducted once every 8 days (totally 6 times) until July 31, 2014 (69 DAP). At each sampling time, one seedling was randomly chosen from each container of N4 treatment (0.105 mg cm^–3^) from each chamber to measure stomatal conductance, transpiration, and photosynthetic rates of the youngest fully expanded leaf between 09:00 and 11:00 by a portable photosynthesis system (Li-6400, LI-COR, United States) with 500 μmol m^–2^ s^–1^ active radiation, 500 mmol s^–1^ air flow rate, and sample cell CO_2_ concentration equal to that in the chamber. Meanwhile, one seedling from all the containers was sampled to measure fresh weight, and the leaves and roots were scanned and analyzed by the WinRHIZO Pro software package (Regent Instruments Inc., QC, Canada) to obtain leaf area, root length, and diameter. For roots and aboveground organs, dry weights were determined by drying for 48 h to a constant at 70°C. Tissue P and K concentrations were, respectively, measured as described in Snell and Snell (1949) and Hao et al. (2016), and tissue N concentration was measured with an element analyzer (CHNSO EA 1108, Carlo Erba reagents, Italy). On 53 DAP (the fourth sampling event), nitrate was extracted with 0.001 mmol cm^–3^ CaSO_4_ solution from fresh samples of the pulverized leaves and roots by using a mortar, followed by centrifugation (Bloom et al., 2014). The ${\text{NO}}_{3}^{-}$-N concentration of the diluted extracts was determined with a flow auto-analyzer (AA3, SEAL Analytical, Germany). Subsequently, tissue ${\text{NO}}_{3}^{-}$-N concentration was estimated and normalized by total N in plant tissues (${\text{NO}}_{3}^{-}$-N/total N), as described in Bloom et al. (2014). Water consumed by transpiration was quantified daily by weighing the containers at 20:00 and considering the change of plant fresh weight.

### Exp. 2: Soil- N × CO_2_

An experiment was conducted in 2019 featuring the culture of winter wheat (*Triticum aestivum* L. cv. Nongda 212) in soil columns, which were 55 cm high and 15 cm in diameter. In total, 63 polyvinyl chloride columns were cleaved vertically into two halves that were then reconnected and sealed with covers at the bottom. All the columns were packed with air-dried sandy soil (93.3% sand, 1.7% silt, and 5.0% clay) upto a height of 50 cm at a bulk density of 1.55 g cm^–3^. The original soil nutrient status was very poor with total N as low as 220.03 mg kg^–1^, available P 4.27 mg kg^–1^, and available K 55.67 mg kg^–1^. Soil water retention was measured with a pressure membrane plate (Soil Moisture Equipment Co., United States) and described using the closed form of van Genuchten (1980) as: saturated soil water content of 0.376 cm^3^ cm^–3^; soil residual water content of 0.010 cm^3^ cm^–3^; the fitted coefficients of α = 0.087 cm^–1^; and *n* = 1.626. Water content at field capacity was chosen as 0.095 cm^3^ cm^–3^ corresponding to soil matric potential of −100 cm (Romano and Santini, 2002).

Wheat seeds were germinated as described for Exp. 1. On February 22, 2019, seedlings were planted with a density of 3 plants per column, similar to typical conditions in the field (400–600 plants m^–2^). The experiment lasted for 92 days until the tillering stage (May 24, 2019). Constant conditions in the two growth chambers were kept as: photosynthetic photon flux density of 500 μmol m^–2^ s^–1^ over plants from 08:00 to 20:00; day/night temperature of 25/15°C ± 1°C; and relative humidity of 40% ± 5%. Before March 20 (27 DAP), all the soil columns were irrigated with half-strength Hoagland solution to keep optimal soil water and nutrient conditions for wheat growth, and the initial CO_2_ concentration in the two chambers was maintained at 400 ± 50 μmol mol^–1^.

On 27 DAP, three soil columns were randomly chosen and sampled to quantify conditions before initiating treatments. After removing the shoots, columns were opened. The soil cores were cut into 5 cm height layers and soil was sampled to measure water content by oven-drying to a constant weight at 105°C, and inorganic N concentration in soil solution was measured with a flow autoanalyzer. The remainder of each soil layer was placed on a screen with 0.05 cm diameter grid and washed to collect the roots. For roots in each soil layer, leaves and stems, parameters such as, leaf area, root length and diameter, fresh and dry weight, tissue concentrations of N, P, and K, were determined as described for Exp. 1. The remaining 60 columns were mulched with 3 cm of fine quartz sand to minimize evaporation and irrigated to field capacity. Fertilizer was applied to each column with P and K contents (the ratio to dried soil) of 62 and 108 mg kg^–1^, respectively. For the 30 columns in each chamber, two N levels were set by applying urea at 90 (named as LN) or 180 mg N kg^–1^ (HN). At 08:00 on 27 DAP, the CO_2_ concentration in one of the two chambers was elevated from 400 ± 50 to 850 ± 50 μmol mol^–1^, and kept unchanged as 400 ± 50 μmol mol^–1^ in the other chamber. Three duplicate columns under each treatment were randomly chosen to measure stomatal conductance and photosynthetic rates of the youngest fully expanded leaves between 10:00 and 14:00, as described for Exp. 1.

To keep optimal soil water conditions for wheat growth (Feddes et al., 1978), irrigation was triggered whenever the average soil water content in the whole column was lower than 80% of field capacity, and terminated when field capacity was reached. Sampling was conducted every 13 days (following irrigation events) 5 times from 40 to 92 DAP. For each sampling event, three duplicate columns were randomly chosen from each treatment to measure the same parameters that were measured on 27 DAP. Three soil columns under each treatment were selected and weighed daily to estimate the corresponding average soil water content and plant transpiration by considering the change of plant fresh weight. Due to mulching, soil evaporation was assumed to be negligible (Shi et al., 2013).

### Exp. 3 Hydroponic- Humidity × N

A hydroponic experiment on winter wheat (*Triticum aestivum* L. cv. Nongda 212), including three atmospheric relative humidity levels, three N supply levels, and two N supply patterns (3 × 3 × 2 treatments), was conducted in 2017. The constant growth conditions in the three chambers were as follows: photosynthetic photon flux density of 400 μmol m^–2^ s^–1^ over plants from 06:00 to 18:00; day/night temperature of 30/20°C ± 1°C; CO_2_ concentration of 400 ± 50 μmol mol^–1^. As described for Exp. 1, wheat seeds were germinated and planted in quartz sand until May 7, 2017 (22 DAP) and then the seedlings were transplanted into 90 containers (28 cm in height, 18 cm in width, and 24 cm in length). Each container was filled with half-strength Hoagland solution and covered with a board that had eight holes (2 cm in diameter) bored uniformly. Each seedling occupied one hole and was fixed with sponge. Before 29 DAP, the relative humidity in the three chambers was kept at 50% ± 10%.

At 08:00 on May 14, 2017 (29 DAP), the air relative humidity in the three growth chambers was adjusted to 50% ± 10%, 70% ± 10%, and 90% ± 10%, respectively. Within each chamber, there were six treatments, a design of three N (NH_4_NO_3_) supply levels (0.007, 0.053, and 0.105 mg cm^–3^ in modified half-strength Hoagland solution) and two N supply patterns (Table 1). In the first supply pattern of “day water–night nutrient” (DW–NN), plants were cultured in deionized water during daytime from 08:00 to 20:00 and moved to nutrient solution during nighttime from 20:00 to 08:00. In the second supply pattern of “day nutrient–night nutrient” (DN–NN), plants were constantly cultured in nutrient solution. Each treatment was replicated five times with a total of 30 containers in each chamber. For the pattern of DW–NN, in order to alternate solution environment conveniently, each container filled with nutrient solution was accompanied by an identical container filled with deionized water. Roots were rinsed three times in deionized water when the plants were moved from containers filled with nutrient solution into those with deionized water. The nutrient solution or deionized water in each container was replaced every 3 days, and fresh air was injected continuously with a compressor.

**TABLE 1:** For each wheat seedling under each treatment with unique N supply pattern (day water–night nutrient, DW-NN; day nutrient–night nutrient, DN-NN) and level and relative humidity in Exp. 3, dry weight of aboveground biomass and its N concentration on 77 days after planting (DAP), transpiration, N uptake mass, and root N uptake factor (*δ*) during the treatment period (29–77 DAP).

N supply patterns	N supply levels (mg cm^–3^)	Relative humidity (%)	Dry weight in aboveground (g)	N concentration in aboveground (mg g^–1^)	Transpiration (× 10^3^ cm^3^)	N uptake (mg)	Root N uptake factor δ
					Daytime + Nighttime	Daytime + Nighttime	
DW-NN	0.007	50%	6.59 ± 0.13d	25.88 ± 1.48h	(2.07+0.39) ± 0.04de	(0+207.63) ± 7.07g	75.52 ± 1.45a
		70%	6.18 ± 0.58e	27.94 ± 1.82g	(1.53+0.36) ± 0.04fg	(0+209.78) ± 25.16g	79.82 ± 3.7a
		90%	6.52 ± 0.71d	26.77 ± 1.89gh	(1.48+0.36) ± 0.02fg	(0+209.96) ± 17.82fg	80.84 ± 0.89a
	0.053	50%	7.71 ± 0.39cd	45.16 ± 1.86e	(2.48+0.43) ± 0.05c	(0+390.98) ± 7.76e	17.25 ± 0.41b
		70%	7.79 ± 0.40cd	43.47 ± 1.26f	(1.68+0.35) ± 0.09f	(0+386.23) ± 20.48e	19.90 ± 0.88b
		90%	7.80 ± 0.30d	47.02 ± 1.22d	(1.65+0.40) ± 0.07f	(0+365.46) ± 31.42e	21.00 ± 1.14b
	0.105	50%	8.66 ± 0.74bc	45.99 ± 1.67de	(2.68+0.57) ± 0.07a	(0+468.23) ± 16.17d	7.78 ± 0.30e
		70%	8.72 ± 0.21bc	54.00 ± 1.15a	(2.09+0.47) ± 0.22de	(0+514.80) ± 24.84cd	10.43 ± 0.47d
		90%	8.93 ± 0.49bc	51.22 ± 0.68bc	(1.91+0.42) ± 0.05e	(0+465.01) ± 40.83d	9.87 ± 0.13d
DN-NN	0.007	50%	7.92 ± 0.34c	25.02 ± 1.10h	2.38 ± 0.12e	217.85 ± 8.5rg	13.01 ± 0.38c
		70%	7.71 ± 0.59c	24.80 ± 1.46h	1.69 ± 0.09h	222.90 ± 15.71fg	18.61 ± 2.18b
		90%	7.77 ± 0.84c	25.77 ± 1.25h	1.42 ± 0.06i	230.66 ± 22.87fg	20.14 ± 2.12b
	0.053	50%	10.05 ± 0.18ab	47.21 ± 1.27d	2.96 ± 0.14b	500.88 ± 9.62cd	3.23 ± 0.11h
		70%	9.33 ± 0.31b	48.68 ± 0.91cd	2.38 ± 0.13e	488.97 ± 13.02cd	4.03 ± 0.13g
		90%	9.72 ± 0.19ab	49.28 ± 1.36c	1.99 ± 0.03f	514.04 ± 8.44c	5.00 ± 0.07f
	0.105	50%	10.56 ± 0.26a	51.85 ± 0.97b	3.08 ± 0.06ab	577.41 ± 16.85d	1.75 ± 0.02j
		70%	10.33 ± 0.31a	49.75 ± 1.19c	2.55 ± 0.13d	539.95 ± 18.15b	1.94 ± 0.10j
		90%	10.61 ± 0.27a	55.50 ± 0.41a	2.08 ± 0.08f	611.52 ± 14.42a	2.68 ± 0.04i

*For the treatment of DW-NN, transpiration and N uptake were, respectively, estimated during daytime and nighttime and root N uptake factor was estimated during nighttime when transpiration and N uptake occurred simultaneously. Different lowercase letters in the same column indicate significant difference among treatments at p < 0.05 tested with one-way ANOVA.*

Sampling was first conducted on 29 DAP and was then repeated every 12 days (5 times in total) until July 1 (77 DAP). At each sampling, as described for Exp. 1, one seedling was randomly chosen from each container to measure fresh weight, leaf area, root length and diameter, dry weights, and tissue N concentrations of roots and aboveground tissues. For the pattern of DN-NN, transpiration consumption was determined daily by weighing the containers at 20:00 and considering the change of plant fresh weight. For the pattern of DW–NN, the containers were weighed twice daily at 08:00 and 20:00 when the solution environment was alternated, and thus transpiration was calculated for both during daytime and nighttime periods.

### Estimating Root N Uptake Factor

In hydroponic Exps. 1 and 3, plant N uptake, transpiration, and inorganic N concentration in nutrient solution were used to calculate a dimensionless root N uptake factor (*δ*) according to a macroscopic root N uptake model (Dalton et al., 1975; Schoups and Hopmans, 2002; Shi et al., 2013; Li S. et al., 2018):


(1) $$\delta =\frac{M_{SU}}{V_{TP}C_{N}}$$

where *M*_*SU*_ is plant N uptake during a period (mg); *V*_*TP*_ is transpiration volume occurring simultaneously with N uptake (cm^3^); and *C*_*N*_ is inorganic N concentration at root surfaces, substituted by the solution N concentration under well-stirred hydroponic conditions (mg cm^–3^). Generally, a value of 0 < *δ* ≤ 1 specifies that only passive uptake exists, whereas *δ* > 1 indicates that passive and active uptake coexist. Moreover, a higher *δ* implies greater dominance of active uptake (Dalton et al., 1975; Schoups and Hopmans, 2002; Ingwersen and Streck, 2005; Li S. et al., 2018).

### Statistical Analysis

Analysis of variance (ANOVA) was performed using the statistical software package SPSS 20.0 (SPSS, United States). With the general linear model (GLM) procedure in the software package, a multifactor ANOVA was conducted to explore the interaction of the continuous variables (e.g., CO_2_ concentration, relative humidity, DAP and N supply level) on the studied parameters such as photosynthesis, transpiration, dry weight, leaf area, tissue N concentration, N uptake, root N uptake factor, etc. Statistical significance was evaluated on the least significant difference (LSD) at a 0.05 probability level. In addition to CO_2_ concentrations in Exps.1 and 2 and air relative humidity in Exp. 3, the other climate conditions in the various growth chambers remained the same for each experiment. Therefore, a one-way ANOVA was adopted to evaluate the effects of CO_2_ concentrations in Exps. 1 and 2 or air relative humidity in Exp. 3 on the studied parameters. Homogeneity of variance was checked before one-way ANOVA was performed. The coefficient of variation for all response variables were below 10%. Graphs were produced using Sigmaplot 14.0 (Systat Software, United States).

## Results

### Plant Growth Affected by CO_2_ and N Supply

**FIGURE 1:**
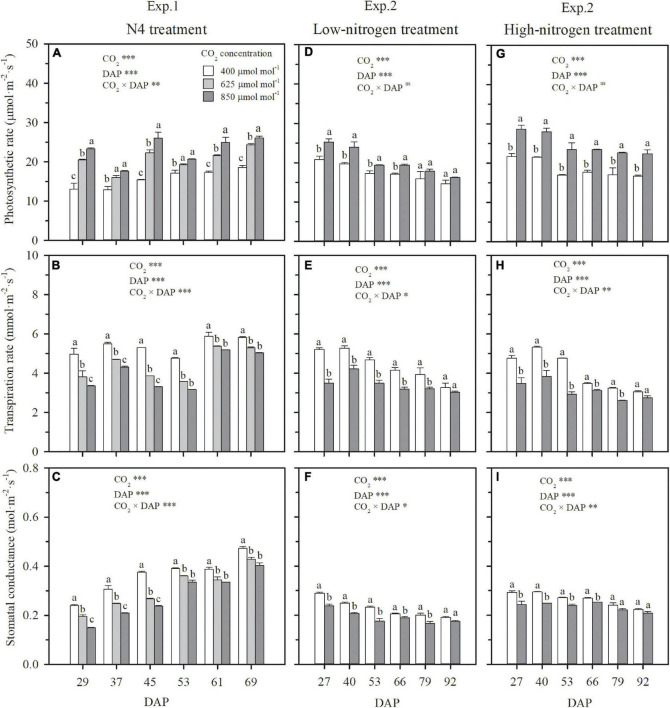
The effect of CO_2_ concentration (400, 625, and 850 μmol mol^–1^) on the photosynthetic rates, transpiration rates, and stomatal conductance of youngest fully expanded wheat leaves when the ${\text{NO}}_{3}^{-}$-N concentration in solution was 0.105 mg cm^–3^ in Exp. 1 **(A–C)**, and under low nitrogen **(D–F)** and high nitrogen **(G–I)** treatments in Exp. 2. Error bars represent standard error of three replications. Different lowercase letters among CO_2_ treatments indicate significant difference at *p* < 0.05 tested with one-way ANOVA. Asterisks indicate the significance level of two-way ANOVA between CO_2_ and DAP and their interaction: ns, no significant difference; *p < 0.05; ^**^p < 0.01; ^***^p < 0.001. DAP: days after planting.

**FIGURE 2:**
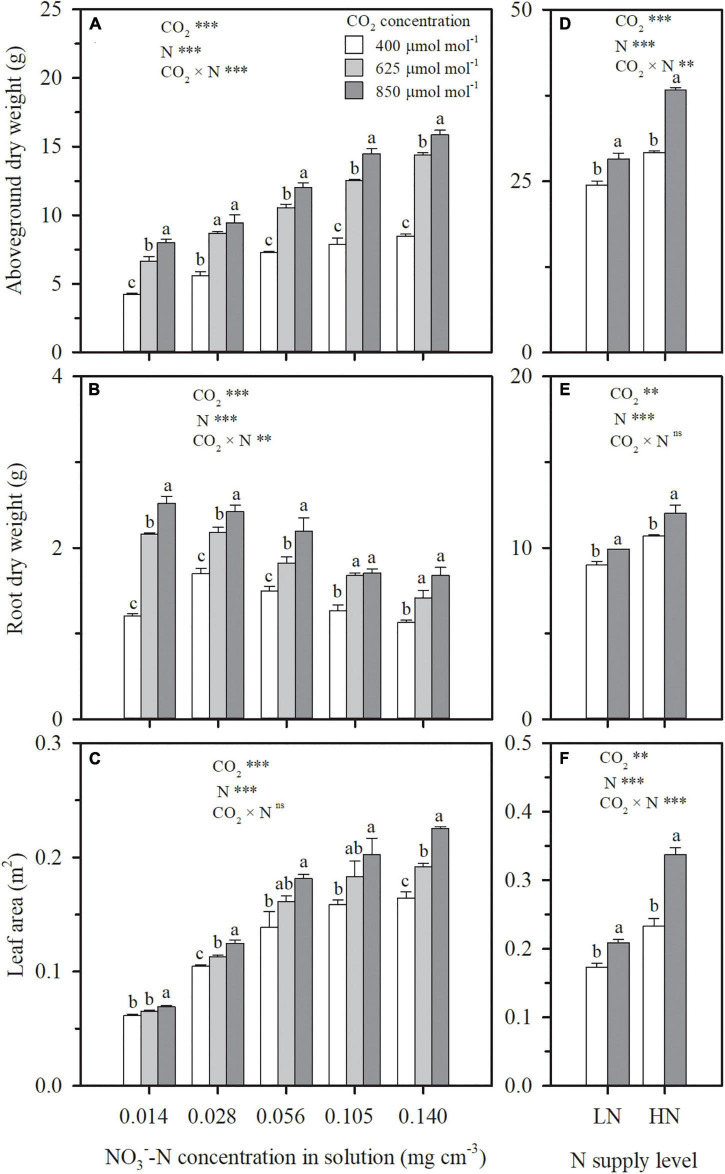
On 69 days after planting in Exp. 1, the effect of CO_2_ concentration (400, 625, and 850 μmol mol^–1^) and solution ${\text{NO}}_{3}^{-}$-N concentration on aboveground dry weight **(A)**, root dry weight **(B),** and leaf area **(C)** per wheat seedling. On 92 days after planting in Exp. 2, the effect of CO_2_ concentration (400 and 850 μmol mol^–1^) and N supply level (low nitrogen, LN; high nitrogen, HN) on aboveground dry weight **(D)**, root dry weight **(E)** and leaf area **(F)** per wheat seedling. Error bars represent standard error of three replications. Different lowercase letters among CO_2_ treatments indicate significant difference at *p* < 0.05 tested with one-way ANOVA. Asterisks indicate the significance level of two-way ANOVA between CO_2_ and N and their interaction: ns represent no significant difference; *P < 0.05; ^**^P < 0.01; ^***^P < 0.001.

Whether wheat was cultured in nutrient solution (Exp. 1) or well-watered soil columns (Exp. 2), elevating CO_2_ concentration stimulated leaf-scale photosynthesis (Figures 1A,D,G) and thus crop growth (Figure 2). The extent of stimulation was dependent on the degree and duration of elevation. Under N4 treatment in Exp. 1 with solution ${\text{NO}}_{3}^{-}$-N at a concentration of 0.105 mg cm^–3^, the photosynthetic rate of the youngest fully expanded leaves during the CO_2_ treatment period (29–69 DAP) was enhanced by 32 and 48% on an average by elevating CO_2_ concentration from 400 to 625 and 850 μmol mol^–1^, respectively (Figure 1A). Consequently, the dry weights of aboveground and root system biomass and leaf area on 69 DAP, were, respectively, raised by 60, 33, and 15% under 625 μmol mol^–1^, and 84, 49, and 28% under 850 μmol mol^–1^ (Figures 2A,C). A significant interaction effect on leaf photosynthetic rate was observed between DAP and CO_2_ concentration (*P* < 0.01), accompanied by a decreasing contribution of e[CO_2_] with the prolongation of time. When wheat was exposed to e[CO_2_] from 29 (the first day) to 69 DAP, a general reduction in the enhancement in leaf photosynthetic rate from 57 to 31% under 625 μmol mol^–1^ and from 78 to 40% under 850 μmol mol^–1^ occurred (Figure 1A). In addition, increase of biomass due to e[CO_2_] was different for various N supply levels and plant organs, and interaction was evident (*p* < 0.05). For example, when solution ${\text{NO}}_{3}^{-}$-N concentration was raised from 0.014 mg cm^–3^ under N1 to 0.140 mg cm^–3^ under N5, compared to ambient CO_2_ concentration (400 μmol mol^–1^), the enhancement of aboveground dry weight under 625 μmol mol^–1^ rose from 58 to 70%, whereas the increase of root dry weight declined from 79 to 26% (Figures 2A,B). This indicated that, when N supply was insufficient, the positive effect of e[CO_2_] was more significant on root system than on aboveground tissues, and that the situation reversed when N supply was sufficient. In general, the contribution of e[CO_2_] on plant growth increased with N supply in both Exps. 1 and 2 (Figure 2), which agreed well with the effects of e[CO_2_] on leaf photosynthetic rate (Figures 1D,G).

### Plant Transpiration Affected by CO_2_ and N Supply

**FIGURE 3:**
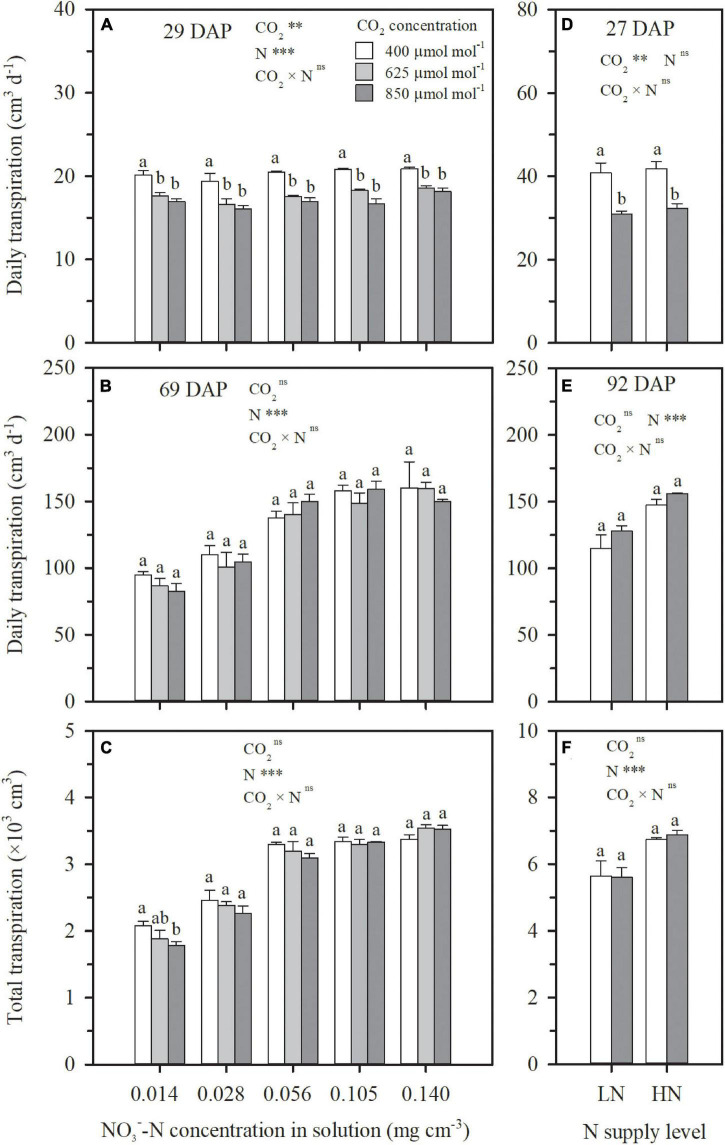
The effect of CO_2_ concentration (400, 625, and 850 μmol mol^–1^) and solution ${\text{NO}}_{3}^{-}$-N concentration on transpiration consumption on 29 DAP **(A)**, 69 DAP **(B),** and 29–69 DAP **(C)** per wheat seedling in Exp. 1. The effect of CO_2_ concentrations (400 and 850 μmol mol^–1^) and N supply level (low nitrogen, LN; high nitrogen, HN) on transpiration on 27 DAP **(D)**, 92 DAP **(E),** and 27–92 DAP **(F)** per wheat seedling in Exp. 2. Error bars represent standard error of three replications. Different lowercase letters among CO_2_ treatments indicate significant difference at *p* < 0.05 tested with one-way ANOVA. Asterisks indicate the significance level of two-way ANOVA between CO_2_ and N and their interaction: ns represent no significant difference; *p < 0.05; ^**^p < 0.01; ^***^p < 0.001. DAP: days after planting.

Under both hydroponic (Exp. 1) and well-watered soil (Exp. 2) conditions, stomatal conductance and leaf transpiration rate were significantly reduced by e[CO_2_], but the reduction weakened or even disappeared over time, a significant interaction effect between CO_2_ concentration and DAP (Figures 1B,C,E,F,H,I). For the N4 treatment in Exp. 1, in comparison to ambient CO_2_ concentration, the reduction of leaf transpiration rates under 625 and 850 μmol mol^–1^ narrowed from 23 and 32% on 29 DAP to 9 and 13% on 69 DAP, respectively (Figure 1B). For HN and LN treatments in Exp. 2, leaf transpiration rates were on average limited by 30% on 27 DAP when CO_2_ concentration was raised from 400 to 850 μmol mol^–1^, but the limitation gradually weakened and became insignificant on 92 DAP with an average reduction of 8% (Figures 1E,H). On the first day of CO_2_ treatment when the difference of leaf area among treatments was negligible, plant-scale transpiration rates were significantly limited by e[CO_2_] under all the N treatments in Exps.1 and 2 (Figures 3A,D), similar to leaf-scale transpiration rates (Figures 1B,E,H). However, due to the interaction between the weakened limitation on leaf transpiration rate (Figures 1B,E,H) and the enlargement of leaf area under e[CO_2_] (Figures 2C,F), the reduction of daily plant transpiration also weakened and then disappeared (Figures 3B,E). Thus, significant influence was not observed on plant total transpiration throughout the CO_2_ treatment periods (29–69 DAP in Exp. 1 and 27–92 DAP in Exp. 2), except for the extreme N deficit treatment of N1 in Exp. 1 with an average decrease of 12% (Figures 3C,F).

### Plant N Status and Uptake Affected by CO_2_ and N Supply

**FIGURE 4:**
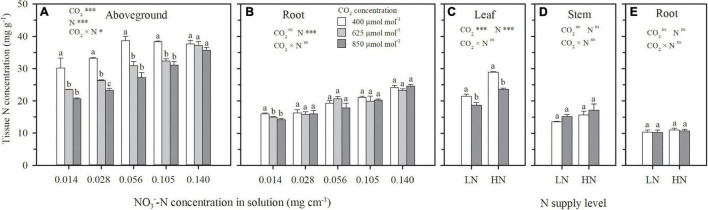
On 69 days after planting in Exp. 1, the effect of CO_2_ concentration (400, 625 and 850 μmol mol^–1^) and solution ${\text{NO}}_{3}^{-}$-N concentration on N concentration in aboveground tissue **(A)** and roots **(B)**. On 92 days after planting in Exp. 2, the effect of CO_2_ concentration (400 and 850 μmol mol^–1^) and N supply level (low nitrogen, LN; high nitrogen, HN) on N concentration in leaves **(C)**, stems **(D),** and roots **(E)**. Error bars represent standard errors of three replications. Different lowercase letters among CO_2_ treatments indicate significant difference at *p* < 0.05 tested with one-way ANOVA. Asterisks indicate the significance level of two-way ANOVA between CO_2_ and N and their interaction: ns represent no significant difference; *p < 0.05; ^**^p < 0.01; ^***^p < 0.001.

Under both hydroponic (Exp. 1) and well-watered soil (Exp. 2) conditions, when N supply was insufficient, e[CO_2_] resulted in N deficiency. On 69 DAP in Exp. 1, ECIND was found in wheat aboveground tissues, except for the sufficient N supply treatment of N5, weakening with the increasing N supply level (Figure 4A). On 92 DAP in Exp. 2, ECIND occurred in leaves under the two N treatments (Figure 4C) although not in the stems (Figure 4D). Only under extreme N deficit conditions such as N1 in Exp. 1, ECIND was found in root systems, with an average 9% decrease in root N concentration (Figures 4B,E). Under ambient CO_2_, aboveground N concentration increased gradually with increasing N supply level from N1 to N3 and then kept stable as N supply continued to increase (Figure 4A). Under e[CO_2_], however, aboveground N concentration increased consistently from N1 to N5, indicating an increase in N demand.

**FIGURE 5:**
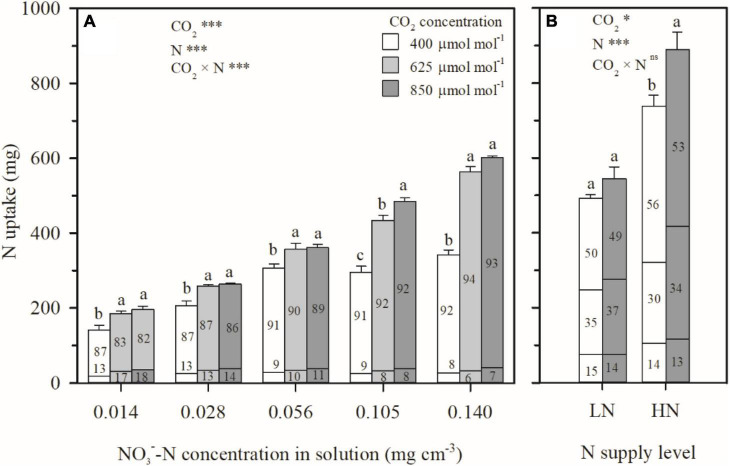
The effect of CO_2_ concentration (400, 625, and 850 μmol mol^–1^) and N supply level on N uptake per wheat seedling between 29–69 days after planting in Exp. 1 **(A)** and 27–92 days after planting in Exp. 2 **(B)**. The N uptake was divided into two parts in Exp. 1 (top of columns: allocated in aboveground biomass; bottom of columns: in roots) and three parts in Exp. 2 (top: allocated in leaves; middle: in stem; bottom: in roots). The numbers in each column represent the percentage of N uptake allocated in the corresponding organs. Error bars represent standard errors of three replications for N uptake. Different lowercase letters among CO_2_ treatments indicate significant difference at *p* < 0.05 tested with one-way ANOVA. Asterisks indicate the significance level of two-way ANOVA between CO_2_ and N and their interaction: ns represent no significant difference; *P < 0.05; ^**^P < 0.01; ^***^P < 0.001.

**FIGURE 6:**
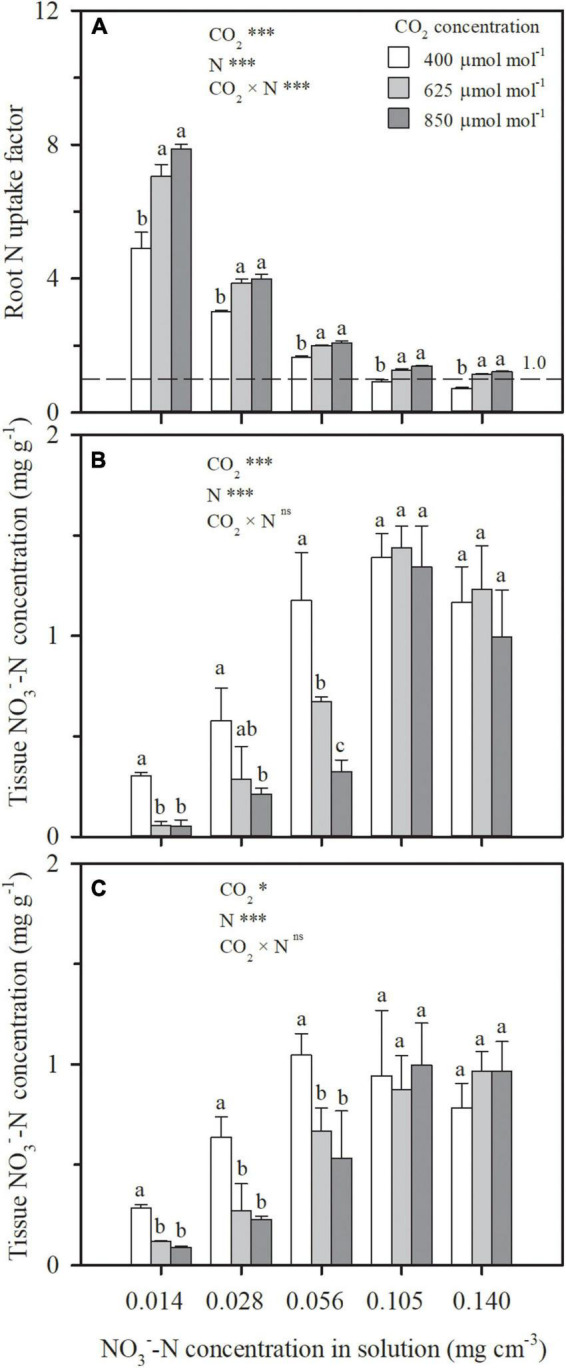
The effect of CO_2_ concentration (400, 625 and 850 μmol mol^–1^) and N supply level on root N uptake factor during 29–69 days after planting **(A)**, and on ${\text{NO}}_{3}^{-}$-N concentration in leaves **(B)** and roots **(C)** of wheat seedlings on 53 days after planting in Exp. 1. Error bars represent standard error of three replications. Different lowercase letters among CO_2_ treatments indicate significant difference at *p* < 0.05 tested with one-way ANOVA. Asterisks indicate the significance level of two-way ANOVA between CO_2_ and N and their interaction: ns represent no significant difference; *p < 0.05; ^**^p < 0.01; ^***^p < 0.001.

Even as e[CO_2_] led to similar or decreased water uptake (Figures 3C,F) and N deficiency (Figures 4A,C), more N uptake was found as a function of increasing CO_2_ concentration under all the N treatments in Exps. 1 and 2 (Figure 5). During the CO_2_ treatment periods, compared to ambient CO_2_ concentration, N uptake under N1–N5 treatments was averagely enhanced by 33, 23, 17, 42, and 69% by e[CO_2_] (Figure 5A), and 10 and 20% under LN and HN, respectively (Figure 5B). Consequently, the root N uptake factor δ was significantly raised by e[CO_2_] in Exp. 1 regardless of N supply levels (Figure 6A), implying more active N uptake. Furthermore, the e[CO_2_]-induced enhancement for δ weakened with increasing N supply level, indicating less increase of active uptake. When solution ${\text{NO}}_{3}^{-}$-N concentration was raised from 0.014 mg cm^–3^ (N1) to 0.140 mg cm^–3^ (N5), the average enhancement of δ under the two e[CO_2_] treatments decreased from 52 to 43%. For each CO_2_ concentration, δ also decreased with increasing N supply level (Figure 6A). Under the N5 treatment, values of δ close to 1.0 indicated dominance of passive uptake, whereas δ values between 5 and 8 under N1 treatment implied that active uptake was around 4–7 times greater than passive uptake.

### The Effects of Plant Transpiration on N Uptake

For any specific combination of N supply pattern and level, when air relative humidity rose from 50 to 70% or even 90%, transpiration was significantly limited, whereas plant biomass, tissue N concentration, and N uptake were almost not affected, resulting in an increase in δ (Table 1). For example, during the treatment period (29–77 DAP) when wheat was supplied with 0.007 mg N cm^–3^ solution through the pattern of DN-NN, increase of air relative humidity from 50 to 70% or 90% reduced plant transpiration by 29 and 40% and caused increases of 24 and 92% in δ, respectively. For any specific combination of N supply level and air relative humidity, relative to DN-NN pattern, plant growth, transpiration, and N uptake under DW–NN treatment were limited, but the corresponding transpiration occurring simultaneously with N uptake was limited much more, thus resulting in a higher value of δ (Table 1). For example, when wheat was cultured with 0.053 mg N cm^–3^ solution and under 70% air relative humidity, the N uptake of each plant seedling during treatment period was 488.97 mg (daytime + nighttime) under DN–NN and 386.23 mg (nighttime) under DW-NN, and the corresponding transpiration was 2.38 and 0.35 × 10^3^ cm^3^, respectively. Relative to DN–NN, N uptake under DW–NN treatment was reduced by only 21%, while the corresponding transpiration was reduced by 85%, leading to an increase of 394% in δ.

### Plant ${\text{NO}}_{3}^{-}$ Assimilation Affected by CO_2_ and N Supply

Similar to tissue N concentration (Figures 4A,B), under each CO_2_ concentration in Exp. 1, ${\text{NO}}_{3}^{-}$-N concentration in leaves or roots first increased and were then kept stable or even decreased as a function of increasing solution ${\text{NO}}_{3}^{-}$ concentration (Figures 6B,C). Relative to ambient CO_2_, ${\text{NO}}_{3}^{-}$ buildup was not found under e[CO_2_], whereas lower ${\text{NO}}_{3}^{-}$-N concentrations were observed in both leaves and roots when solution ${\text{NO}}_{3}^{-}$-N concentration was less than 0.105 mg cm^–3^. When ${\text{NO}}_{3}^{-}$-N was normalized by total N in plant tissues (${\text{NO}}_{3}^{-}$-N/total N) for each N supply level, similar trend was found (data not shown), indicating that plant ${\text{NO}}_{3}^{-}$ assimilation was not inhibited by e[CO_2_].

## Discussion

### Relationship Between ECIND and Reduced Transpiration

Except for the sufficient N supply treatment of N5 in Exp. 1, ECIND was found in aboveground organs, especially in leaves under both hydroponic and well-watered soil conditions (Figures 4A,C), agreeing well with many findings for various plant species, varieties, and environmental conditions (Ainsworth and Rogers, 2007; Bloom et al., 2010; Halpern et al., 2019). Before discussing the relationship between plant transpiration and N uptake, it is important to note that the effect of e[CO_2_] on transpiration itself was not straightforward. At first, a decrease in both leaf and plant-scale transpiration rates under e[CO_2_] was found in Exps. 1 and 2 (Figures 1B,E,H, 3A,D). However, the decline in leaf transpiration rate caused by e[CO_2_] was duration dependent and, by the end of the experiments, the effect was much smaller or insignificant. This is in agreement with the conclusions of Medlyn et al. (2001) and Leakey et al. (2006), who found that the effect of e[CO_2_] on stomatal conductance declines over time alongside the effect of e[CO_2_] on photosynthesis. On the other hand, leaf area itself was positively affected by e[CO_2_] (Figures 2C,F). The interaction between leaf transpiration rate and leaf area led to unchanged transpiration per plant due to e[CO_2_] at the end of Exps. 1 and 2 (Figures 3B,E) or throughout the CO_2_ treatment periods (Figures 3C,F), with the exception of the lowest N supply treatment (N1) in Exp. 1 resulting from the lower increase of leaf area (Figure 2C).

It is interesting that in Exps. 1 and 2, more N was taken up by plants under e[CO_2_], regardless of N supply levels compared to ambient CO_2_ concentration (Figure 5), in spite of similar or decreased transpiration (Figures 3C,F) and N deficiency (Figures 4A,C). This agrees well with the previous findings for wheat and other C3 plants (Kanemoto et al., 2009; Houshmandfar et al., 2018). The link between N uptake and transpiration was directly tested in Exp. 3 by manipulating transpiration through relative humidity and by discriminating between times at which N uptake and transpiration occurred. The results from Exp. 3 indicated that, at least under hydroponic conditions with sufficient N supply to root surfaces, root N uptake might be uncoupled from water uptake, and decrease in plant transpiration would not necessarily lead to a reduction in N uptake, except under the extreme condition such as DW–NN treatment where N was supplied during only nighttime rather than daytime (Table 1). This should be attributed to the physiological mechanisms (Dalton et al., 1975; Schoups and Hopmans, 2002; Shi et al., 2013), supporting plants to more actively taken up N to satisfy N demand under e[CO_2_] in Exps. 1 and 2 or when transpiration was directly limited as in Exp. 3, with a higher root N uptake factor (*δ*) in the macroscopic root N uptake model (Figure 6A and Table 1). Under well-watered conditions with sufficient N transport from the surrounding media to root surfaces, changes in transpiration therefore do not account for ECIND. The question remains however, with the compensation mechanism of active uptake, why do plants under hydroponic conditions (e.g., DW–NN treatment in Exp. 3) fail to completely make up for reduced N uptake due to extreme decreases in transpiration? Similarly, why cannot plants, under hydroponic conditions or well-watered soil conditions with low N availability, actively take up more N to avoid ECIND even when transpiration is not reduced?

Whether transpiration is reduced as in Exp. 3 or plant growth is accelerated by e[CO_2_] as in Exps. 1 and 2, the difference between passive uptake and plant N demand increases. When passive uptake fails to satisfy N demand, active uptake, dependent on energy consumption by plants, will play a role (Dalton et al., 1975; Ingwersen and Streck, 2005; Li S. et al., 2018). Even under well-watered conditions, active uptake may be limited due to the need to expend energy and therefore cannot always eliminate the gap between passive uptake and N demand. The higher the plant N demand, or the less the passive uptake due to lower N availability or transpiration, the bigger the gap, i.e., more serious N stress and a greater need to expend energy in order to increase active uptake, as is seen in the increasing root N uptake factor as a function of e[CO_2_] or reduced N supply level and transpiration (Figure 6A and Table 1) and as discussed in the literature (Dalton et al., 1975; Schoups and Hopmans, 2002; Shi et al., 2013). The limited compensation of active uptake due to energy consumption reasonably explains N dilution under e[CO_2_]. Following this line of reasoning, increasing N supply might be a useful method to relieve or even avoid ECIND (Sims et al., 1998; Kim et al., 2001; Dier et al., 2018), although it is sometimes economically or environmentally unfeasible.

### Relationship Between ECIND and N Dilution/Resource Allocation

Under e[CO_2_], due to the limited compensation of active uptake, when the increase of N uptake cannot match the increase of carbohydrates as the ratio under ambient CO_2_ concentration, N dilution occurs in plant tissues and accounts for ECIND (Monje and Bugbee, 1998; Stitt and Krapp, 1999; Halpern et al., 2019). Typically, tissue N concentration decreases with plant growth. Relative to the initial tissue N concentration before initiating treatments (29 DAP in Exp. 1 and 27 DAP in Exp. 2), the ratio of the increased N mass to the increased dry weight in aboveground or leaves (ΔN/ΔDW, mg g^–1^) during the CO_2_ treatment periods always decreased under each treatment (Table 2). Under the N5 treatment in Exp. 1 with sufficient N supply, CO_2_ concentration did not significantly impact ΔN/ΔDW, and thus ECIND did not occur (Figure 4A). However, under the other treatments in Exps. 1 and 2, compared to ambient CO_2_ concentration, e[CO_2_] led to lower values of ΔN/ΔDW and greater decrease relative to initial tissue N concentration before initiating treatments (Table 2), agreeing well with the e[CO_2_]-induced decrease in tissue N concentration (Figures 4A,C). Generally, the lower value of ΔN/ΔDW resulted in more serious N deficiency. This result strongly validated the argument for dilution as an explanatory effect for ECIND. Besides N, dilution under e[CO_2_] was also found for P and K elements in both Exps.1 and 2, with a decrease of tissue concentration within 2–19%, agreeing well with the finding for tomato (Halpern et al., 2019).

**TABLE 2:** Under each treatment, the ratio of increased N mass to dry weight in aboveground biomass during 29–69 days after planting (DAP) in Exp. 1 and leaves during 27–92 DAP in Exp. 2 (ΔN/ΔDW), the corresponding change percentage to the tissue N concentration on 29 and 27 DAP, as well as the root/shoot ratios of N mass (R/S_N) and dry weight (R/S_DW) on 69 and 92 DAP, respectively.

Treatments		400				625				850			
		ΔN/ΔDW (mg g^–1^)	Change percentage (%)	R/S_N	R/S_DW	ΔN/ΔDW (mg g^–1^)	Change percentage (%)	R/S_N	R/S_DW	ΔN/ΔDW (mg g^–1^)	Change percentage (%)	R/S_N	R/S_DW
Exp. 1	N1	29.88 ± 3.24	−29.7	0.15 ± 0.01a	0.33 ± 0.01a	23.24 ± 0.14	−45.33	0.22 ± 0.01a	0.30 ± 0.03a	20.40 ± 0.30	−52.01	0.21 ± 0.01a	0.30 ± 0.01a
	N2	33.02 ± 0.32	−22.32	0.15 ± 0.02a	0.31 ± 0.01b	26.16 ± 0.17	−38.44	0.16 ± 0.01b	0.25 ± 0.02b	23.10 ± 0.57	−45.71	0.16 ± 0.01b	0.24 ± 0.01b
	N3	38.60 ± 1.36	−9.19	0.10 ± 0.01b	0.20 ± 0.02c	30.84 ± 1.27	−27.44	0.11 ± 0.01c	0.17 ± 0.01c	27.10 ± 1.56	−36.25	0.11 ± 0.02c	0.18 ± 0.01c
	N4	38.28 ± 0.27	−9.93	0.10 ± 0.01b	0.16 ± 0.01d	32.30 ± 0.67	−24	0.08 ± 0.01d	0.13 ± 0.01d	31.04 ± 1.08	−26.97	0.09 ± 0.01d	0.12 ± 0.01d
	N5	37.57 ± 1.11	−11.61	0.09 ± 0.01b	0.13 ± 0.01e	37.15 ± 1.21	−12.6	0.06 ± 0.01e	0.10 ± 0.01e	35.66 ± 0.81	−16.09	0.07 ± 0.01e	0.11 ± 0.01d
Exp. 2	LN	29.17 ± 0.32	−50.56	0.17 ± 0.01a	0.38 ± 0.01a	–	–	–	–	24.17 ± 0.16	−59.05	0.17 ± 0.01a	0.34 ± 0.01a
	HN	29.62 ± 0.93	−49.8	0.17 ± 0.01a	0.37 ± 0.01a	–	–	–	–	23.24 ± 0.30	−**60**.62	0.15 ± 0.01b	0.31 ± 0.02b

*Different lowercase letters in the same column indicate significant difference among treatments in each experiment at P < 0.05 tested with one-way ANOVA.*

When N uptake is insufficient to keep up with growth, plants will adjust resource allocation to minimize N limitation (Coleman et al., 1993; Kanemoto et al., 2009). Compared to the high N treatment (N5) in Exp. 1, with the decreasing solution ${\text{NO}}_{3}^{-}$-N concentration, N uptake was lowered as a higher ratio than that of plant dry weight, regardless of CO_2_ concentration. For example, under the condition of 850 mmol mol^–1^ CO_2_ concentration, N uptake during 29–69 DAP under N4, N3, N2, and N1 treatments decreased by 7%, 40%, 56%, and 67% on average (Figure 5A), whereas the total plant dry matter weight decreased by 7, 19, 32, and 45%, respectively (Figures 2A,B). Although more resources were allocated in root system with higher root/shoot ratios of N mass or dry weight (Table 2), tissue N concentrations in both aboveground (Figure 4A) and root system (Figure 4B) decreased under the low N treatments (N1–N4). Photosynthetic product (Figures 2A,B) and N uptake (Figure 5A) were promoted under e[CO_2_] regardless of N supply level. For any treatment among N1, N2, N3, and N4, where the increment in N uptake could not match the increment in photosynthetic product as the ratio under ambient CO_2_ concentration, N was preferentially allocated to root system by adjusting the root/shoot ratios of N mass and dry weight (Table 2), to keep an almost stable root N concentration (Figure 4B), resulting in N deficiency in the aboveground parts (Figure 4A). This result was validated in Exp. 2 under well-watered soil conditions (Figures 2D,E, 4C,D,E, 5B). This physiological response benefits plants under e[CO_2_] to take up more N by maintaining relatively high root uptake activity (Shi et al., 2013) and to prevent more serious N deficiency in the way of photosynthetic acclimation (McGrath and Lobell, 2013; Butterly et al., 2015; Ruiz-Vera et al., 2017).

### Relationship Between ECIND and Reduced ${\text{NO}}_{3}^{-}$ Assimilation

Another hypothesis explaining ECIND was proposed by Bloom et al. (2002, 2010, 2014). Through a large variety of methods, they demonstrated that e[CO_2_] decreases ${\text{NO}}_{3}^{-}$ assimilation due to decreased photorespiration. As a result, ${\text{NO}}_{3}^{-}$ is accumulated in plant tissues and, in turn, limits ${\text{NO}}_{3}^{-}$-N uptake and leads to N deficiency. However, evidence of e[CO_2_]-induced reduction of ${\text{NO}}_{3}^{-}$ assimilation was not found for any ${\text{NO}}_{3}^{-}$-N supply level in hydroponic Exp. 1, where the ${\text{NO}}_{3}^{-}$-N concentrations (Figures 6B,C) or the ratios of ${\text{NO}}_{3}^{-}$-N/total N in both leaves and roots were similar or lower than those under ambient CO_2_ concentration. This result validated the previous findings (Kruse et al., 2002; Jauregui et al., 2016; Halpern et al., 2019; Adavi and Sathee, 2021). More N uptake under e[CO_2_] (Figure 5) implied that ${\text{NO}}_{3}^{-}$ assimilation should be accelerated rather than limited, due to the enhanced activity (Kruse et al., 2002; Jauregui et al., 2016) or transcript level (Stitt and Krapp, 1999; Adavi and Sathee, 2021) of ${\text{NO}}_{3}^{-}$ reductase under e[CO_2_]. Under the N1, N2, and N3 treatments in Exp. 1, the lower tissue ${\text{NO}}_{3}^{-}$-N concentration induced by e[CO_2_] should result from the greater N demand and the insufficient N supply (Figures 6B,C). The similar tissue ${\text{NO}}_{3}^{-}$-N concentration under the N4 and N5 treatments should be attributed to the sufficient N supply. The different results regarding ${\text{NO}}_{3}^{-}$ assimilation under e[CO_2_] might be related to time, conditions, and methods of tissue ${\text{NO}}_{3}^{-}$-N concentration measurement, a compound which is known to be highly transient (Koch et al., 1988).

## Conclusion

Under hydroponic or well-watered soil conditions, compared to ambient CO_2_ concentration, e[CO_2_] resulted in similar or less transpiration consumption of wheat during periods of elevated CO_2_, and was accompanied by increased N uptake and higher tissue dry weight, as well as lower N concentration in aboveground organs in cases where N was supplied at deficit rates. Due to the compensation caused by active uptake, N uptake may be uncoupled from water uptake under well-watered conditions, and thus changes in transpiration did not account for ECIND. Decreased assimilation of ${\text{NO}}_{3}^{-}$ was not found under e[CO_2_] and could therefore also be eliminated as a major cause of ECIND under our conditions. The effect of dilution, stemming from the fact that increased N uptake under e[CO_2_] failed to match the increase of carbohydrates, comes forward as a feasible and dominant mechanism explaining ECIND under these experimental conditions. In addition to dilution, ECIND could also be at least partially attributed to adjustment in resource allocation as the physiological responses to e[CO_2_], through which stable root N concentration was consistently kept. These results, especially the effects of e[CO_2_] on ${\text{NO}}_{3}^{-}$ assimilation, should be further investigated and validated under various soil, plant, and climate conditions.

## Data Availability Statement

The raw data supporting the conclusions of this article will be made available by the authors, without undue reservation.

## Author Contributions

JShi designed the study. JF, YY, YF, and MH collected and analyzed the data. JF and JShi wrote the manuscript. All authors reviewed the manuscript.

## Conflict of Interest

The authors declare that the research was conducted in the absence of any commercial or financial relationships that could be construed as a potential conflict of interest.

## Publisher’s Note

All claims expressed in this article are solely those of the authors and do not necessarily represent those of their affiliated organizations, or those of the publisher, the editors and the reviewers. Any product that may be evaluated in this article, or claim that may be made by its manufacturer, is not guaranteed or endorsed by the publisher.

## Footnotes

1
http://co2now.org
